# *ELK3*: A New Molecular Marker for the Diagnosis and Prognosis of Glioma

**DOI:** 10.3389/fonc.2021.608748

**Published:** 2021-12-16

**Authors:** Zhendong Liu, Zhishuai Ren, Cheng Zhang, Rongjun Qian, Hongbo Wang, Jialin Wang, Wang Zhang, Binfeng Liu, Xiaoyu Lian, Yanbiao Wang, Yuqi Guo, Yanzheng Gao

**Affiliations:** ^1^ Department of Surgery of Spine and Spinal Cord, Henan Provincial People’s Hospital; People’s Hospital of Zhengzhou University, People’s Hospital of Henan University, Zhengzhou, China; ^2^ People’s Hospital of Zhengzhou University, Henan Provincial People’s Hospital, Zhengzhou, Henan, China; ^3^ North Broward Preparatory School, Nord Anglia Education, Coconut Creek, FL, United States; ^4^ Department of Neurosurgery, Henan Provincial People’s Hospital, Zhengzhou, China; ^5^ People’s Hospital of Henan University, Henan Provincial People’s Hospital, Zhengzhou, China; ^6^ Department of Neurosurgery, The First Affiliated Hospital of Harbin Medical University, Harbin, China; ^7^ Department of Obstetrics and Gynecology, Henan Provincial People’s Hospital, People’s Hospital of Zhengzhou University, People’s Hospital of Henan University, Zhengzhou, China; ^8^ Henan International Joint Laboratory for Gynecological Oncology and Nanomedicine, Zhengzhou, China

**Keywords:** glioma, ELK3, biomarker, oncology, prognosis

## Abstract

ETS transcription factor ELK3 *(ELK3)*, a novel oncogene, affects pathological processes and progression of many cancers in human tissues. However, it remains unclear whether *ELK3*, as a key gene, affects the pathological process of gliomas and the prognosis of patients with gliomas. This study aimed to comprehensively and systematically reveal the correlation between *ELK3* and the malignant progression of gliomas by analyzing clinical sample information stored in multiple databases. We revealed the putative mechanism of *ELK3* involvement in malignant gliomas progression and identified a new and efficient biomarker for glioma diagnosis and targeted therapy. Based on the sample data from multiple databases and real-time quantitative polymerase chain reaction (RT-qPCR), the abnormally high expression of *ELK3* in gliomas was confirmed. Kaplan-Meier and Cox regression analyses demonstrated that a high *ELK3* expression was markedly associated with low patient survival and served as an independent biomarker of gliomas. Wilcox and Kruskal-Wallis tests revealed that expression of *ELK3* was positively correlated with several clinical characteristics of patients with gliomas, such as age, WHO classification, and recurrence. Moreover, Cell Counting Kit‐8 (CCK-8), immunofluorescence, and wound healing assays confirmed that *ELK3* overexpression markedly promoted the proliferation and migration of glioma cells. Finally, gene set enrichment analysis (GSEA) and western blotting confirmed that overexpression of *ELK3* regulated the JAK–STAT signaling pathway and upregulate the expression of signal transducer and activator of transcription 3 (STAT3) and phosphorylated STAT3 (P-STAT3) to promote the malignant transition of gliomas. Therefore, *ELK3* may serve as an efficient biomarker for the diagnosis and prognosis of gliomas and it can also be used as a therapeutic target to improve the poor prognosis of patients with gliomas.

## Introduction

Gliomas, a special type of tumor derived from glial cells of the central nervous system, are the most common type of primary malignant cancer in the brain, accounting for approximately 81% of malignant brain tumors (1). The World Health Organization (WHO) has classified the malignancy of gliomas according to anaplastic features into WHO grades I to IV. Grade III and IV gliomas are considered high-grade gliomas, and their extremely poor prognosis is associated with huge social and medical burdens (2). After receiving surgical treatment, radiotherapy, and chemotherapy, patients with high-grade gliomas can attain delayed tumor progression, and these treatments prolong survival. Unfortunately, high-grade gliomas exhibit invasive growth, high recurrence, and extremely complex pathological processes, and the average survival time of patients with gliomas is only 15 months due to the lack of specific biomarkers for diagnosis and individualized treatment (3, 4). Tumor biomarkers can be used for screening, differential diagnosis, and clinical staging of cancer. They can also be used to evaluate tumor volume and response to treatment as prognostic indicators of disease progression (5). Although various biomarkers related to gliomas are used in clinical practice, such as O6-methylguanine-DNA methyltransferase (MGMT) and osteopontin (OPN), their sensitivity and specificity are debated (6, 7). Hence, there is an urgent need for a highly specific and sensitive biomarker for gliomas identification, early diagnosis, and molecular-targeted therapy to prolong the survival of patients with gliomas.

*ELK3*, also known as NET, SAP-2, or ERP, contains the ETS domain. After the formation of the ternary complex factor, this gene can regulate the expression of specific DNA sequences, including those of proto-oncogenes, thus changing the biological behaviour of tumor cells (8, 9). Several prior studies have found that *ELK3* is overexpressed in cancer cells and is involved in tumor cell transfer, angiogenesis, and malignant evolution (10–14). Kim et al. have reported that abnormal expression of *ELK3* from lymphatic endothelial cells can promote the progression and metastasis of breast cancer by regulating microRNAs (15). Furthermore, Lee et al. have observed that *ELK3* was upregulated in hepatocellular carcinoma and promoted the migration and invasion of hepatocellular carcinoma cells (11). Additionally, Wang et al. have reported that *ELK3* is involved in the promotion of stemness and drug resistance in colorectal cancer cells (16). Mao et al. have revealed that silencing the expression of *ELK3* can inhibit the migration of prostate cancer cells (17). Although *ELK3* is well known to be related to cell transfer and invasion and plays a crucial role in the occurrence or evolution of malignant tumors, no research on *ELK3* in gliomas has been reported. Thus, the impact of *ELK3* on the occurrence and progression of gliomas remains unclear.

Therefore, based on the analysis of large numbers of samples from multiple databases, we explored, for the first time, the correlations of *ELK3* with the clinical features of gliomas as well as the pathological process and the potential mechanism of *ELK3*’s involvement in multi-level gliomas. This research provides a new direction for research on the pathological mechanisms of gliomas and a new target biomarker for the diagnosis and prognosis of gliomas.

## Materials and Methods

### Data Sources

The Gene Expression Profiling Interactive Analysis (GEPIA, http://gepia.cancer-pku.cn/index.html) database is a professional interactive web application for gene expression analysis. The data on tumor samples and non-tumor samples were obtained from the Cancer Genome Atlas (TCGA) and the genotype-tissue expression (GTEx) databases, which are recognized as valuable, time-saving resources for researchers (18). We used GEPIA, a powerful online database, to evaluate the expression of *ELK3* in human tumor tissues. Data from 163 glioblastoma (GBM) samples, 518 brain lower grade gliomas (LGG) samples, and 207 normal brain tissue samples were obtained from the database.

The Gene Expression Omnibus (GEO, https://www.ncbi.nlm.nih.gov/geo/) stores data from approximately 20,000 studies from approximately 8000 laboratories worldwide, representing approximately 500,000 samples. Through this database, genes can be retrieved and processed through multiple mechanism levels (19). To study the expression of *ELK3* in gliomas, we obtained two datasets from the GEO database: GSE4290 and GSE50161. GSE4290 included 77 gliomas tissue samples and 23 normal brain tissue samples, and GSE50161 included 34 gliomas tissue samples and 13 normal brain tissue samples. The limma package of R software (v3.6.1) was used to evaluate the differential expression of *ELK3* between gliomas tissues and normal brain tissues. The cut-off criteria were P < 0.05 and log FC > 1.

The Human Protein Atlas (HPA, https://www.proteinatlas.org/) represents the most extensive and systematic database of the spatial distribution of proteins in tissues and cells and is a valuable resource for exploring expression models in specific diseases (20). We analyzed data on *ELK3* expression at the protein level in normal brain tissue and gliomas tissue.

TCGA (https://portal.gdc.cancer.gov/) is a research network containing abundant gene and tumor data and is used to analyze the molecular aberrations and functional effects of various tumors, providing a foundation for tumor treatment (21). We obtained data on 653 gliomas samples from the TCGA RNA-seq database. Clinical information of the patients is shown in **Table S1**.

The Chinese Glioma Genome Atlas (CGGA, http://www.cgga.org.cn/) was established by the Beijing Tiantan Hospital, which is affiliated with the Capital Medical University. The database covers approximately 2000 samples of different types of gliomas, including CGGA RNA-seq data and CGGA microarray data (22). We obtained data on 748 CGGA RNA-seq gliomas samples and 268 CGGA microarray gliomas samples from the database. The clinical information of the corresponding patients is presented in **Tables S2** and **S3**.

### Patients and Tissue Preparation

10 cases of gliomas and 10 cases of non-glioma brain tissue were frozen rapidly in liquid nitrogen within 15 minutes after operation. All patients signed written informed consent. The research program was approved by the Ethics Committee of People’ s Hospital of Henan Provincial.

### Cell Culture and Transfection

The human astrocyte cell line (HA) and gliomas cell line (U251) were obtained from Wuhan Procell Biotechnology Co., Ltd., China. The cells were cultured in Dulbecco’s modified eagle medium (DMEM) high glucose medium (Procell, Wuhan, China) containing 10% fetal bovine serum (Gibco, USA) and 1% penicillin–streptomycin mixture (Procell, Wuhan, China) and placed in a humidified cell incubator at 37°C and 5% CO_2_. The transfection method used in this study was a transient transfection. U251 cells were seeded in 6-well plates, and each well was transfected with 100 pmol siRNA using siRNA-Mate (GenePharma, Shanghai, China) transfection reagent. Serum-free medium was used for transfection, and the complete medium was replaced after 6 h. Transfection efficiency was determined by RT-qPCR at 36 h after transfection. The siRNA sequence with the highest transfection efficiency was selected for subsequent experiments.

### Total RNA Extraction and RT-qPCR

Extract total RNA from cells or tissues using RNA extraction kit (Omega-Biotek) according to the manufacturer’s instructions, and cDNA was obtained using NovoScript Plus All-in-one 1st Strand cDNA Synthesis SuperMix (gDNA Purge) (Novoprotein, E047), and RT-qPCR was performed using NovoStart SYBR qPCR SuperMix Plus (Novoprotein, E096). The primer sequences of *ELK3* and internal reference gene *18S* were purchased from Henan Shangya Biotechnology Co., Ltd. RNA-specific primer sequences for the internal reference gene *18S* were as follows: forward, 5’-GTAACCCGTTGAACCCCATT-3’ and reverse, 5’-CCATCCAATCGGTAGTAGCG -3’. The specific primer sequences of the target gene *ELK3* were as follows: forward, 5’-ATCTGCTGGACCTCGAACGA-3’ and reverse, 5’-TTCTGCCCGATCACCTTCTTG -3’.

### Cell Counting Kit‐8 (CCK-8) Assay

The transfected cells were seeded in a 96-well plate (2000 cells/well). After a 12 h incubation, 110 μL of complete medium containing 10 μL was added to each well and incubated at 37°C for 3 h. The absorbance of the cell solution was measured using a microplate reader at 450 nm wavelength. The results of the first measurement were used as the results of 0 h, based on which the absorbance of cells after CCK-8 addition was measured at 24, 48, and 72 h after culture.

### Immunofluorescence Staining

The transfected U251 cells and control U251 cells were seeded in cell culture dishes and cultured in a cell culture dish for 48 h. The medium was discarded, and the cells were fixed with 4% paraformaldehyde solution at room temperature for 10 min. Subsequently, 0.1% Triton X-100 diluted with PBS was used to penetrate the cells at room temperature for 10 min. After washing with PBS, the Ki-67 primary antibody was added and incubated at room temperature for 1 h (1:200, Abcam, China). After incubation with the primary antibody, cells were washed with PBS three times for 5 min each time. DyLight 594 IgG (1:200, Invitrogen, USA) fluorescent antibody was incubated in the dark at room temperature for 1 h. Nuclei were stained with DAPI for 10 min. Finally, the expression levels of Ki-67 in the experimental and control groups were compared using a fluorescence microscope.

### Wound Healing Assay

The transfected cells and control cells were inoculated into 6-well plates. Three replicate wells were used for the parallel experiments for each cell. When the cells reached 90% density, each hole was scratched with a 200 μL sterile pipette tip, and then the cells were washed three times with 1× PBS to remove the exfoliated cells. An appropriate amount of serum-free medium was added to each well. At 40× magnification, the scratches were photographed with a phase contrast microscope as the experimental data for 0 h, and then the cells were cultured in an incubator at 37°C. After 48 h, the scratches were photographed at the same position as in the previous photograph.

### Gene Set Enrichment Analysis (GSEA)

GSEA is a powerful bioinformatics tool for evaluating whether a set of previously defined genes (for example, genes in common signaling pathways) has significant and consistent significant differences between two biological phenotypes (23). Here, a list of genes was generated, ordered according to the correlation of expression with *ELK3* expression, and then GSEA was performed to clarify the significant differences in cell signaling pathways between the high *ELK3* expression and low *ELK3* expression groups. Each analysis required 1000 gene set permutations. The expression level of *ELK3* was then used as a phenotypic marker. NES > 1.80, P < 0.05, and FDR q-val < 0.25 were used as cut-off criteria.

### Western Blotting

Untreated U251 cells were used as the control group, and transfected U251 cells were used as the experimental group. After the cells were full, the total protein was extracted, separated by SDS-PAGE, and electrotransferred to a PVDF membrane. The cells were incubated overnight at 4°C with the following primary antibodies: anti-JAK2 antibody (1:500, Proteintech), anti-P-JAK2 antibody (1:500, Affinity), anti-STAT3 antibody (1:500, Proteintech), and anti-P-STAT3 antibody (1:500, Affinity). After incubation with HPR-labeled secondary antibody (1:10000, Abcam) for 1 h, immunoreaction was detected by enhanced chemiluminescence.

### The Connectivity Map Database (CMap) Analysis

The Connectivity Map database (CMap, https://portals.broadinstitute.org/cmap/) is a gene expression profile database based on intervention gene expression, and it is mainly used to reveal the functional relationships among small-molecule compounds, genes, and diseases (24). First, we used Pearson correlation coefficient analysis to identify the top 10 positively and the top 10 negatively correlated genes of *ELK3* based on the RNA-seq dataset of 748 gliomas samples in the CGGA database. Subsequently, we uploaded the positively correlated genes as upregulated genes (P < 0.05, correlation coefficient > 0.8) and the negatively correlated genes as downregulated genes (P < 0.05, correlation coefficient< −0.40) to the CMap database to identify small-molecule drugs with inhibitory effects on the expression of *ELK3*. Finally, we obtained the 2D and 3D structures and molecular formulas of small-molecule drugs from the PubChem database (https://pubchem.ncbi.nlm.nih.gov/).

### Statistical Analysis

All the data used in this study were processed and analyzed using the R software (v.3.6.1 version). Wilcoxon or Kruskal–Wallis tests were used to analyze the correlations between *ELK3* expression and clinical features. Kaplan–Meier and Cox regression analyses were used to investigate the effect of *ELK3* on the prognosis of gliomas patients and whether *ELK3* has diagnostic value. Univariate and multivariate Cox analyses were used to analyze correlations between clinical parameters and patient prognosis. Pearson correlation coefficient analysis was used to identify genes co-expressed with *ELK3*. The Graphpad Prism software (version 8.0) was used to analyze the difference in *ELK3* expression between the control group and the experimental group. Statistical significance was set at P < 0.05.

## Results

### Elevation of *ELK3* Expression in Gliomas

**Figure 1:**
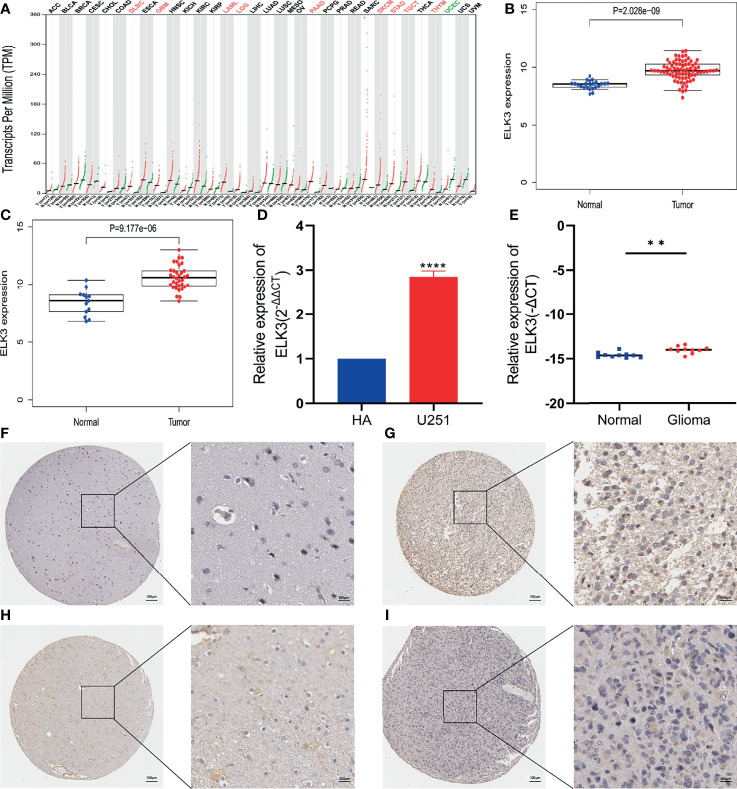
The relative expression of *ELK3* in glioma cells and tissues. **(A)** Based on the GEPIA database, the expression of *ELK3* in various malignant tumors including GBM and LGG. The red marker represents high expression and the green marker represents low expression. **(B)** Based on the GSE 4290 dataset in GEO database, the relative mRNA expression of *ELK3* in glioma tissues and normal brain tissues. **(C)** Based on GSE 50161 dataset in GEO database, the relative mRNA expression of *ELK3* in glioma tissues and normal brain tissues. **(D)** Based on RT-qPCR, the relative expression of *ELK3* in HA cells and U251 cells. **(E)** Based on RT-qPCR, the relative expression of *ELK3* in normal brain tissue samples and glioma tissue samples. **(F–I)** Based on the HPA database, the relative protein expression of *ELK3* in normal brain tissue samples and glioma tissue samples. **P < 0.01, ****P < 0.0001.

First, based on the GEPIA database, we found that *ELK3* is abnormally highly expressed in lymphoid neoplasm diffuse large B-cell lymphoma (DLBC), acute myeloid leukemia (LAML), pancreatic adenocarcinoma (PAAD), skin cutaneous melanoma (SKCM), Stomach adenocarcinoma (STAD), testicular germ cell tumors (TGCT), thymoma (THYM), and other tumor tissues, including GBM and LGG (**Figure 1A**). Second, analysis based on two datasets from the GEO database, GSE4290 and GSE50161, indicated that *ELK3* expression was also higher in gliomas than in normal brain tissues (**Figures 1B, C** **)**. Third, the results based on the HPA database revealed that the protein expression level of *ELK3* in gliomas tissues was markedly higher than that in normal brain tissues (**Figures 1F–I**). Among them, **Figure 1F** is normal brain tissue sample, patient ID is 1539, **Figure 1G** is low grade glioma tissue sample, patient ID is 34, **Figure 1H** is normal brain tissue sample, patient ID is 2523, **Figure 1I** is high grade glioma tissue sample, patient ID is 46. **Figures 1F–I** was stained with HPA001600 antibody. Finally, RT-qPCR results suggested that *ELK3* expression in gliomas cells was markedly higher than that in normal astrocytes (**Figure 1D**), and the expression level in gliomas tissue samples was also markedly higher than that in normal brain tissues (**Figure 1E**). Based on multiple databases and experimental verification, *ELK3* is abnormally highly expressed in gliomas and may be a promoting factor in the development of gliomas.

### Abnormal Expression of *ELK3* Is Strongly Correlated With Poor Prognosis in Patients With Gliomas

**Figure 2:**
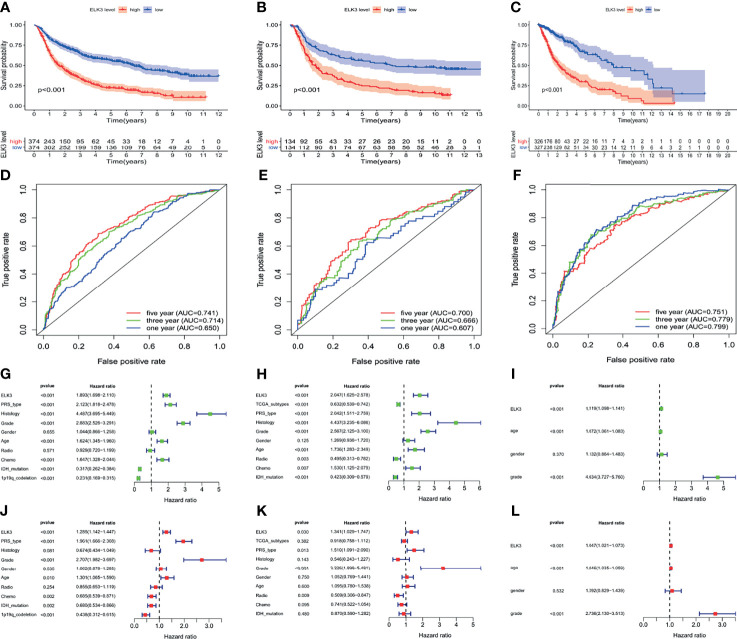
The relationship between different expression patterns of *ELK3* and the prognosis of glioma patients. **(A–C)** Based on the CCGA RNA-seq database, the CCGA microarray database, the TCGA RNA-seq database respectively, the relationship between the different expression patterns of *ELK3* and the prognosis of glioma patients. **(D–F)** Based on the CCGA RNA-seq database, CCGA microarray database, and the TCGA RNA-seq database respectively, the univariate analysis was used to evaluate the risk factors of glioma. **(G–I)** Based on CCGA RNA-seq database, CCGA microarray database, and TCGA RNA-seq database respectively, multivariate analysis was used to evaluate the independent risk factors of glioma. **(J–L)** Based on the CCGA RNA-seq database, the CCGA microarray database, and the TCGA RNA-seq database respectively, the prognostic value of *ELK3* overexpression in glioma patients was evaluated.

First, based on multiple databases, we found that *ELK3* overexpression was closely related to the poor prognosis of patients with gliomas, based on CGGA RNG-seq (P < 0.001, **Figure 2A**), CGGA microarray (P < 0.001, **Figure 2B**), and TCGA RNA-seq (P < 0.001, **Figure 2C**) databases.

Second, based on the CGGA RNA-seq database, the univariate regression analysis revealed that *ELK3* (P < 0.001, [HR] = 1.893 [95% CI [1.698–2.110]), PRS type (P < 0.001, [HR] = 2.123 [95% CI [1.818–2.478])), histology (P < 0.001, [HR] = 4.487 [95% CI [3.695–5.449]), grade (P < 0.001, [HR] = 2.883 [95% CI [2.526–3.291]), age (P < 0.001, [HR] = 1.624 [95% CI [1.345–1.960])), and chemotherapy status (P < 0.001, [HR] = 1.647–952.044) were risk factors for poor prognosis of patients with gliomas (**Figure 2D**). Based on the CGGA microarray database, the univariate regression analysis indicated that *ELK3* (P < 0.001, [HR] = 2.047 [95% CI [1.625–2.578]), PRS type (P < 0.001, [HR] = 2.042 (95% CI [1.511–2.759])), histology (P < 0.001, [HR] = 4.437 [95% CI [3.235–6.086]), grade (P < 0.001, [HR] = 2.567 [95% CI [2.125–3.100]), age (P < 0.001, [HR] = 1.736 [95% CI [1.283–2.349]), and chemotherapy status (P < 0.001, [HR] = 1.530 [95% CI [1.125–2.079])) were risk factors for poor prognosis of patients with gliomas (**Figure 2E**). Based on the TCGA RNA-seq database, the univariate regression analysis indicated that *ELK3* (P < 0.001, [HR] = 1.119 [95% CI [1.098–1.141]), age (P < 0.001, [HR] = 1.072 (95% CI [1.061–1.083])), and grade (P < 0.001, [HR] = 4.634 (95% CI [3.727–5.760]) were risk factors for poor prognosis in gliomas patients (**Figure 2F**). Based on the CGGA RNA-seq database, the multivariate regression analysis revealed that *ELK3* (P < 0.001, [HR] = 1.285 [95% CI [1.142–1.447]), PRS type (P < 0.001, [HR] = 1.961 [95% CI [1.666–2.308])), grade (P < 0.001, [HR] = 2.707 [95% CI [1.982–3.697)), and age (P < 0.001, [HR] = 1.301 [95% CI [1.065–1.590]) were independent risk factors for poor prognosis in gliomas patients (**Figure 2G**). Based on the CGGA microarray database, multivariate regression analysis indicated that *ELK3* (P = 0.030, [HR] = 1.341 [95% CI [1.029–1.747])), PRS type (P = 0.013, [HR] = 1.510 [95% CI [1.091–2.090])), and grade (P < 0.001, [HR] = 3.226 [95% CI [1.898–5.481])) could be used as independent risk factors for poor prognosis of gliomas patients (**Figure 2H**). Based on the TCGA RNA-seq database, the multivariate regression analysis revealed that *ELK3* (P < 0.001, [HR] = 1.047 [95% CI [1.021–1.073]), age (P < 0.001, [HR] = 1.046 (95% CI [1.035–1.059])), and grade (P < 0.001, [HR] = 2.736 [95% CI [2.130–3.513])) were independent risk factors for poor prognosis in gliomas patients (**Figure 2I**).

Finally, we explored the predictive value of *ELK3* for the prognosis of patients with gliomas using receiver operating characteristic curve (ROC) analysis. The ROC analysis based on the CGGA RNA-seq database revealed that the area under the curve (AUC) of 1-, 3-, and 5-year survival rates were 0.741, 0.714, and 0.650, respectively (**Figure 2J**). The ROC analysis based on the CGGA microarray database revealed that the AUC of 1-, 3-, and 5-year survival rates were 0.700, 0.666, and 0.607, respectively (**Figure 2K**). The ROC analysis based on the TCGA RNA-seq database revealed that the AUC of 1-, 3-, and 5-year survival rates were 0.751, 0.779, and 0.799, respectively (**Figure 2L**). The results based on different databases suggested that *ELK3* had a reliable value in predicting the prognosis of glioma patients.

### Correlations Between High Expression of *ELK3* and Clinical Features in Patients With Gliomas

**Figure 3:**
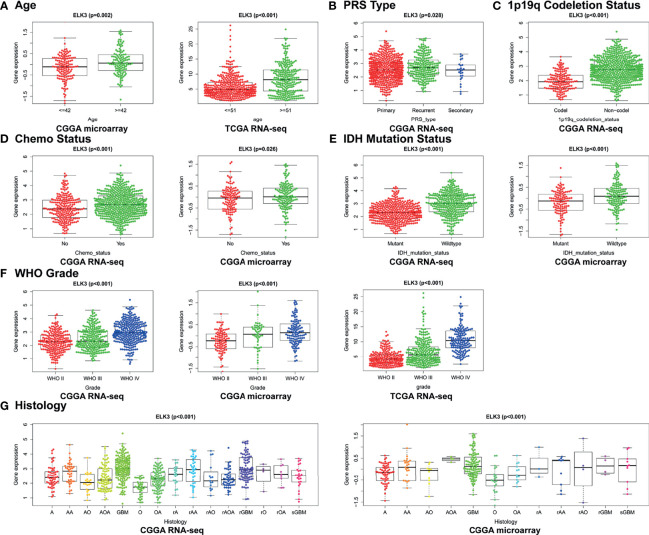
Correlation between *ELK3* expression and clinical features of glioma patients. **(A)** Age. **(B)** PRS types. **(C)** 1p19q codeletion status. **(D)** Chemotherapy status. **(E)** IDH mutation status. **(F)** WHO grade. **(G)** Histology.

The relationships between abnormally high expression of *ELK3* and the clinical characteristics of patients with gliomas were elucidated using the CGGA RNA-seq, CGGA microarray, and TCGA RNA-seq databases. The results are shown in **Figure 3**. In the CGGA RNA-seq database, the expression level of *ELK3* was closely related to WHO grade (P < 0.001), 1p19q codeletion status (P < 0.001), chemotherapy status (P < 0.001), PRS type (P = 0.028), IDH mutation status (P < 0.001), and histology (P < 0.001). In the CGGA microarray database, the expression level of *ELK3* was closely related to WHO grade (P < 0.001), age (P = 0.002), chemotherapy status (P = 0.026), histology (P < 0.001), and IDH mutation status (P < 0.001). In the TCGA RNA-seq database, the expression level of *ELK3* was closely related to WHO grade (P < 0.001) and age (P < 0.001). All these associations were significant.

### Knockdown of *ELK3* Expression Inhibits Proliferation and Migration of Glioma Cells

**Figure 4:**
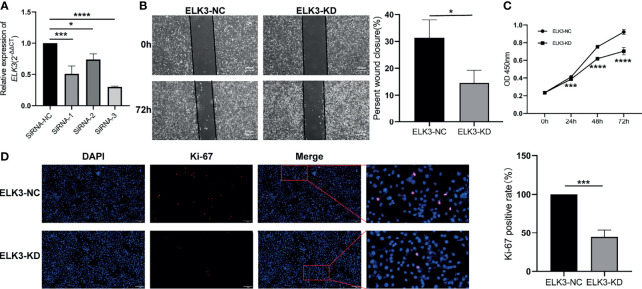
The effect of knocking down the expression of *ELK3* in glioma cells on the proliferation and migration of glioma cells. **(A)** Comparison of knock-down efficiency of siRNA sequences at different sites on *ELK3* expression. **(B)** Scratch healing experiment compared the effect of *ELK3* expression knock-down on glioma cell migration. **(C)** CCK-8 assay was used to compare the effects of *ELK3* expression knock-down on the proliferation of glioma cells. **(D)** Immunofluorescence assay was used to compare the effects of *ELK3* expression knock-down on the proliferation of glioma cells. *P < 0.05, ***P < 0.001, ****P < 0.0001.

Through RT-qPCR, we screened for the optimal siRNA sequence that could target the reduction of *ELK3* expression. To observe the effect of *ELK3* knockdown on the proliferation and migration of glioma cells, we used the screened siRNA sequence to target the downregulation of *ELK3* expression in U251 cells (**Figure 4A**). The CCK-8 assay demonstrated that after the expression of *ELK3* was reduced, the proliferation ability of U251 cells was markedly reduced (**Figure 4C**), which was also confirmed by immunofluorescence analysis of Ki-67 (**Figure 4D**). Moreover, the migration ability of U251 cells was markedly decreased after the expression of *ELK3* was reduced, and a significant difference was observed between the groups (**Figure 4B**). Hence, overexpression of *ELK3* markedly promoted the proliferation and migration of gliomas cells.

### Effects of Different Expression Patterns of *ELK3* in Gliomas Cells on JAK2 - STAT3 Pathway Proteins

**Figure 5:**
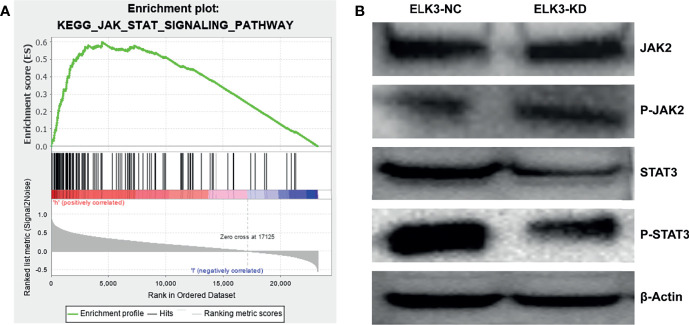
The association between *ELK3* and JAK-STAT signaling pathway in glioma. **(A)** *ELK3* was markedly enriched in JAK-STAT signaling pathway based on GSEA. **(B)** The effect of *ELK3* knockdown on the expression of JAK2, P-JAK2, STAT3 and P-STAT3 in glioma cells by Western blotting.

To explore the mechanism by which *ELK3*, as an oncogene, affects the pathological process and malignant progression of gliomas, we used GSEA to identify the possible signaling pathways of *ELK3* in gliomas. The JAK–STAT signaling pathway exhibited evidently differential enrichment (**Figure 5A**). To further demonstrate how *ELK3* regulates these pathways to affect the malignant progression of gliomas, we explored the regulation of JAK2/STAT3 by *ELK3* through western blot experiments. The results demonstrated that after targeted knockdown of *ELK3* expression in U251 cells, compared with the control group, no significant difference was observed in JAK2 and P-JAK2 expressions, while STAT3 and P-STAT3 expressions were markedly decreased (**Figure 5B**).

### CMap Analysis Results

**Figure 6:**
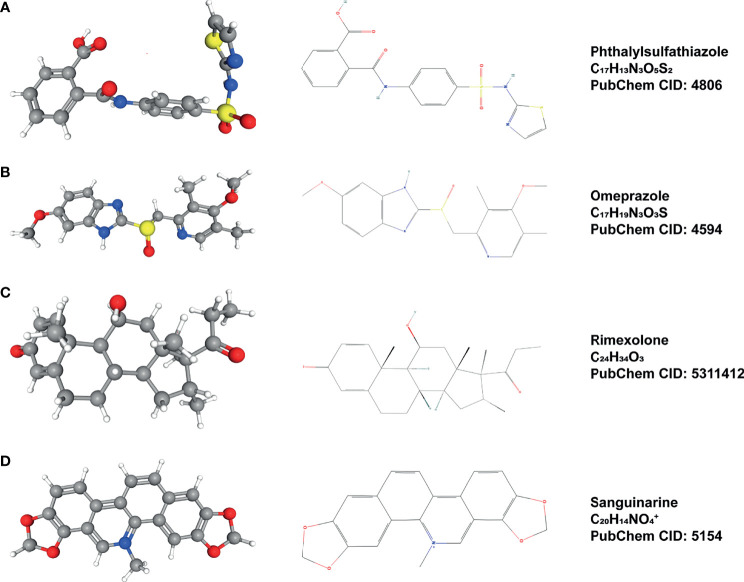
Small-molecule compounds that may inhibit *ELK3* and their 2D and 3D structures. **(A)** Phthalylsulfathiazole. **(B)** Omeprazole, **(C)** Rimexolone, **(D)** Sanguinarine.

Through co-expression analysis, we screened out the top 10 genes with synergistic and inhibitory relationship with *ELK3* expression (**Figure S1** and **Table S4**). Based on the results of co-expression analysis and CMap online tool, we obtained four small-molecule compounds that may have potential inhibitory effects on *ELK3* expression: sanguinarine, omeprazole, rimexolone, and phthalylsulfathiazole. The relevant parameters are presented in **Table 1**. Using PubChem (https://pubchem.ncbi.nlm.nih.gov/), the 2D and 3D structures of these small-molecule compounds were determined (**Figure 6**).

**Table 1:** Small molecule compounds predicted by CMap.

No.	CMap name	Enrichment	P
1	Phthalylsulfathiazole	-0.880	0.00008
2	Omeprazole	-0.888	0.00036
3	Rimexolone	-0.852	0.00092
4	Sanguinarine	-0.971	0.00169

Enrichment <-0.8, p<0.05. CMap, connectivity map.

## Discussion

Many studies have reported that *ELK3* is overexpressed in a variety of cancer cells and involved in tumor cell metastasis, angiogenesis and malignant evolution (10–14). However, whether *ELK3* affects the pathological process of gliomas has not been elucidated. Therefore, our work aimed to explore the expression level of *ELK3* in gliomas and its potential diagnostic and therapeutic value in gliomas.

Using the GEPIA database, we found that *ELK3* was abnormally overexpressed in gliomas compared with normal brain tissue. The expression of *ELK3* in gliomas was verified based on the analysis results of GEO database and HPA database and RT-qPCR results. A previous study shows that *ELK3* is upregulated in hepatocellular carcinoma and enhances the migration and invasion of hepatocellular carcinoma cells (11). Therefore, we speculated that *ELK3* might be involved in the pathological process of gliomas as an oncogene and aimed to reveal the relationship between *ELK3* expression and gliomas prognosis.

Here, abnormally high expression of *ELK3* in gliomas clinical samples was associated with reduced survival time in gliomas patients, as evidenced by analyses using multiple databases. Hence, we generated ROC of *ELK3* expression in gliomas to analyze its prognostic value in gliomas. The results demonstrated that *ELK3* expression had a prognostic value for 1-year, 3-year, and 5-year survival rates in patients with gliomas, with abnormally high expression associated with reduced survival. The univariate and multivariate analyse using Cox regression revealed that high *ELK3* expression might be an independent risk factor for poor diagnosis and prognosis of gliomas. Previous studies have reported that the survival time of patients with gliomas is affected by the expression of target genes, which provides the opportunity to identify new molecular diagnostic and prognostic markers for gliomas (25, 26). Thus, it is plausible that *ELK3* acts as a carcinogenic gene in the progression of gliomas and that high expression of *ELK3* may be an independent risk factor for poor diagnosis and prognosis of patients with gliomas.

To study whether changing the expression pattern of *ELK3* will change the proliferation and migration ability of gliomas cells, we targeted knockdown expression of *ELK3* in U251 cells using cell transfection technology. The results revealed that the proliferation and migration ability of U251 cells decreased after the expression of *ELK3*. As IDH mutation was firstly discovered in diffuse gliomas in 2009, the treatment in diffuse gliomas has gained an increasing prominent role due to its key significance in the diagnosis, prognosis, and prediction of gliomas (27–29). The discovery and key role of IDH mutations also provide important guidance for targeted therapy and vaccine development for patients with gliomas. The high correlation between *ELK3* and the malignant phenotype of gliomas revealed in our study may be of great significance for gene-targeted therapy of gliomas.

In our evaluation of whether synergistic effects occur between abnormally high expression levels of *ELK3* and clinical features related to the prognosis of gliomas, the results suggested that *ELK3* expression was closely related to age, WHO grade, recurrence status, 1p/19q codeletion status, IDH mutation status, and other clinical characteristics of patients with gliomas. The higher the WHO grade, the higher the degree of malignancy of the gliomas (30). Additionally, 1p/19q codeletion and IDH mutation status are associated with chemotherapy sensitivity and good prognosis of gliomas. *ELK3* expression is reportedly higher in 1p/19q non-codeletion and IDH wild-type gliomas than in other gliomas types, suggesting that *ELK3* expression is positively correlated with the malignant degree of gliomas (30, 31). Therefore, it is plausible that *ELK3*, as an oncogene, is closely related to adverse clinical features of gliomas. Consequently, we intend to determine the mechanism by which *ELK3* participates in the pathological process and malignant progression of gliomas.

To explore the specific mechanism of *ELK3* as an oncogene in the progression of gliomas, we explored the cell signaling pathways that *ELK3* may participate in gliomas through GSEA, and investigated its functions. GSEA analysis showed that *ELK3* might promote the malignant progression of gliomas through JAK-STAT signaling pathway. We also conducted related studies by western blotting to explore the regulation mode of *ELK3* on JAK2–STAT3 expression in gliomas. Compared with the control group, after the expression of *ELK3* was knocked down, the expression of STAT3 and P-STAT3 was decreased in the experimental group, while the expression of JAK2 and P-JAK2 was not markedly changed. STAT3, a cytoplasmic transcription factor, activates genes in human chromosome 12 (q13 to q14-1) through phosphorylation of the SH2 domain tyrosine 705. These target genes are involved in various key cellular biological processes, including differentiation, immunity, inflammation, increment, and apoptosis (32, 33). Mis-regulation of STAT3 leads to dysregulation of gene expression patterns, thereby promoting the malignant progression of tumors (34). Many studies have reported that STAT3 plays a key role in the occurrence and development of malignant tumors, especially gliomas (35–37). P-STAT3 can activate the expression of downstream genes, regulate the proliferation, growth, differentiation, and migration of tumor cells, and is closely related to the occurrence, development, and prognosis of gliomas (38–43). Moreover, recent studies have suggested that STAT3 and P-STAT3 may play similar functions in various tumors, including gliomas (44–48). Based on above results, we speculated that *ELK3* overexpression might promote the malignant growth, proliferation, and migration of gliomas through regulating expression of JAK2 and STAT3, especially STAT3. Whether *ELK3* promotes malignant progression of glioma requires further study.

We not only studied the role of *ELK3* as a carcinogenic gene in the occurrence and development of gliomas, but also identified four small-molecule compounds with inhibitory effects on *ELK3*: sanguinarine, omeprazole, rimexolone, and phthalylsulfathiazole. It has been reported that these small-molecule compounds are closely related to the treatment of gliomas. For example, sanguinarine can induce autophagic and apoptotic cell death in gliomas cell lines, providing a new treatment for malignant gliomas (49). Additionally, omeprazole, an aromatic hydrocarbon ligand, can inhibit the invasion and metastasis of breast cancer cells (50).

Here, although we used multiple well-known databases to explain the expression pattern of *ELK3* in gliomas and its relationship with multiple malignant characterizations of gliomas, and verified it experimentally, there are still some unavoidable limitations. Firstly, the clinical samples in the CGGA database we used are all Asian, while the clinical samples in the TCGA database are from different countries, which will affect uniformity in sample sources and may lead to racial differences. However, considering the impact of race on gliomas, our study used a database containing different ethnic samples for analysis and comparison to ensure the comprehensiveness and reliability of the results. Secondly, we used the CMap tool to identify four small-molecule drugs that might be used in gliomas treatment; however, not all of these drugs have been studied for cancer treatment, so their reliability may be questioned. However, previous studies have evaluated the reliability of CMap tools for drug prediction (51, 52). Furthermore, substantial progress has been made in recent years in the field of drug repositioning. Metformin is often used in the treatment of type 2 diabetes because of its hypoglycemic effect. However, Elgendy et al. have recently revealed the inhibitory effect of metformin on tumors and the underlying mechanism (53). Moreover, omeprazole is often used in the treatment of gastric and duodenal ulcers because of its effective inhibition of gastric acid secretion. However, Jin et al. have reported that omeprazole, an aromatic hydrocarbon ligand, can inhibit the invasion and metastasis of breast cancer cells (50). Hence, these drugs may play significant roles in the treatment of gliomas.

## Conclusion

This study is the first to analyze and discover that *ELK3* is abnormally high in gliomas and closely related to the poor prognosis of patients with gliomas comprehensively and systematically. *In vitro* experiments reveal that *ELK3* overexpression can increase the proliferation and migration of gliomas cells and play a role through the regulation of JAK2 the STAT3 signaling pathway. The results provide a valuable candidate target marker for improving the poor prognosis of patients with gliomas and can inform future research on the pathogenesis of gliomas.

## Data Availability Statement

The datasets presented in this study can be found in online repositories. The names of the repository/repositories and accession number(s) can be found in the article/**Supplementary Material**.

## Author Contributions

ZL, ZR, and CZ wrote the draft and revised it. RQ, HW, JW, WZ, BL, XL, and YW performed the data collection, designed the tables and figures. YQG and YZG finally reviewed the manuscript. All authors contributed to the article and approved the submitted version.

## Funding

This work was supported by the Thousand Talents Plan of Central Plains (ZYQR201912122).

## Conflict of Interest

The authors declare that the research was conducted in the absence of any commercial or financial relationships that could be construed as a potential conflict of interest.

## Publisher’s Note

All claims expressed in this article are solely those of the authors and do not necessarily represent those of their affiliated organizations, or those of the publisher, the editors and the reviewers. Any product that may be evaluated in this article, or claim that may be made by its manufacturer, is not guaranteed or endorsed by the publisher.
