# Supplementary figures and images for: ELK3: A New Molecular Marker for the Diagnosis and Prognosis of Glioma
Source: Front Oncol. 2021 Dec 16;11:608748. doi: 10.3389/fonc.2021.608748 (PMC8716454; doi:10.3389/fonc.2021.608748)

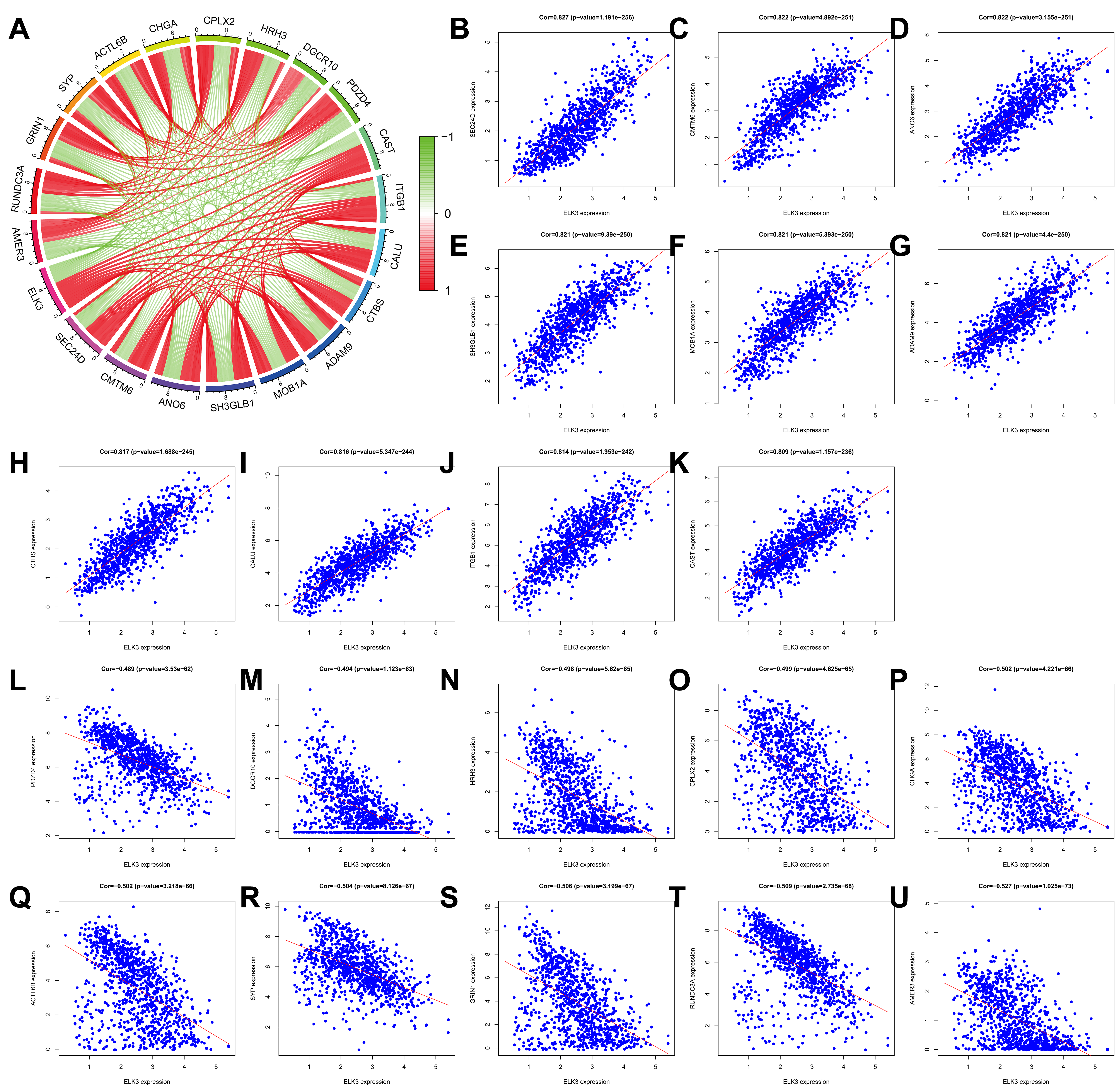

Supplement: Supplementary file 1 [file Image_1.tif]
