# Supplementary material for: ELK3: A New Molecular Marker for the Diagnosis and Prognosis of Glioma
Source: Front Oncol. 2021 Dec 16;11:608748. doi: 10.3389/fonc.2021.608748 (PMC8716454; doi:10.3389/fonc.2021.608748)
Supplement: Supplementary file 3 [file Table_2.docx]

**TABLE S2 |** Characteristics of patients with glioma based on CGGA microarray data

| **Characteristics** |  | **Number of Cases** | **Percentages (%)** |
| --- | --- | --- | --- |
| Gender | Male | 153 | 57.09 |
|  | Female | 115 | 42.91 |
| Age | <=42 | 138 | 51.49 |
|  | >42 | 130 | 48.51 |
| Grade | WHO II | 100 | 37.31 |
|  | WHO III | 52 | 19.40 |
|  | WHO IV | 116 | 43.28 |
| PRS_type | Primary | 238 | 88.81 |
|  | Recurrent | 20 | 7.46 |
|  | Secondary | 10 | 3.73 |
| Radio_status | Yes | 240 | 89.55 |
|  | No | 28 | 10.45 |
| Chemo_status | Yes | 145 | 54.10 |
|  | No | 123 | 45.90 |
| Histology | Astrocytoma | 63 | 23.51 |
|  | Anaplastic astrocytoma | 25 | 9.33 |
|  | Anaplastic oligodendroglioma | 10 | 3.73 |
|  | Anaplastic oligoastrocytoma | 4 | 1.49 |
|  | Glioblastoma | 102 | 38.06 |
|  | Oligodendroglioma | 21 | 7.84 |
|  | Oligoastrocytoma | 13 | 4.85 |
|  | Relapse astrocytoma | 3 | 1.12 |
|  | Relapse anaplastic astrocytoma | 9 | 3.36 |
|  | Relapse anaplastic oligodendroglioma | 4 | 1.49 |
|  | Relapse glioblastoma | 4 | 1.49 |
|  | Secondary relapse glioblastoma | 10 | 3.73 |
| IDH_mutation_status | Mutant | 121 | 45.15 |
|  | Wildtype | 147 | 54.85 |
