# Supplementary material for: ELK3: A New Molecular Marker for the Diagnosis and Prognosis of Glioma
Source: Front Oncol. 2021 Dec 16;11:608748. doi: 10.3389/fonc.2021.608748 (PMC8716454; doi:10.3389/fonc.2021.608748)
Supplement: Supplementary file 4 [file Table_3.docx]

**TABLE S3** | Characteristics of patients with glioma based on TCGA RNA-seq data

| **Characteristics** |  | **Number of Cases** | **Percentages (%)** |
| --- | --- | --- | --- |
| Gender | Male | 377 | 57.73 |
|  | Female | 276 | 42.27 |
| Age | <=51 | 394 | 60.34 |
|  | >51 | 259 | 39.66 |
| Grade | WHO II | 238 | 36.45 |
|  | WHO III | 256 | 39.20 |
|  | WHO IV | 159 | 24.35 |
