# Supplementary material for: ELK3: A New Molecular Marker for the Diagnosis and Prognosis of Glioma
Source: Front Oncol. 2021 Dec 16;11:608748. doi: 10.3389/fonc.2021.608748 (PMC8716454; doi:10.3389/fonc.2021.608748)
Supplement: Supplementary file 5 [file Table_4.docx]

**TABLE S4** | The correlation between genes and *ELK3* based on co-expression analysis

| **Gene Name** | **Correlation Coefficient** | ***P*-value** |
| --- | --- | --- |
| *SEC24D* | 0.827 | 1.19E-256 |
| *CMTM6* | 0.822 | 4.89E-251 |
| *ANO6* | 0.822 | 3.15E-251 |
| *SH3GLB1* | 0.821 | 9.39E-250 |
| *MOB1A* | 0.821 | 5.39E-250 |
| *ADAM9* | 0.821 | 4.40E-250 |
| *CTBS* | 0.817 | 1.69E-245 |
| *CALU* | 0.816 | 5.35E-245 |
| *ITGB1* | 0.814 | 1.95E-242 |
| *CAST* | 0.809 | 1.16E-236 |
| *PDZD4* | -0.494 | 3.53E-62 |
| *DGCR10* | -0.489 | 1.12E-63 |
| *HRH3* | -0.498 | 5.62E-65 |
| *CPLX2* | -0.499 | 4.63E-65 |
| *CHGA* | -0.502 | 4.22E-66 |
| *ACTL6B* | -0.502 | 3.22E-67 |
| *SYP* | -0.504 | 8.13E-67 |
| *GRIN1* | -0.506 | 3.20E-67 |
| *RUNDC3A* | -0.509 | 2.73E-68 |
| *AMER3* | -0.527 | 1.03E-73 |
